# Disturbances in Resting State Functional Connectivity in Schizophrenia: A Study of Hippocampal Subregions, the Parahippocampal Gyrus and Functional Brain Networks

**DOI:** 10.3390/diagnostics15151955

**Published:** 2025-08-04

**Authors:** Raghad M. Makhdoum, Adnan A. S. Alahmadi

**Affiliations:** 1 Department of Radiologic Sciences, Faculty of Applied Medical Sciences, King Abdulaziz University, Jeddah, Saudi Arabia; rmakhdoum0004@stu.kau.edu.sa; 2 Diagnostic Radiology Department, King Abdullah Medical Complex, Jeddah, Saudi Arabia; 3 NMR Research Unit, Department of Neuroinflammation, Queen Square MS Centre, UCL Queen Square Institute of Neurology, London WC1N 3BG, UK

**Keywords:** schizophrenia, hippocampal subregions, parahippocampal gyrus, functional connectivity

## Abstract

**Background/Objectives:** Schizophrenia exhibits symptoms linked to the hippocampus and parahippocampal gyrus. This includes the entorhinal cortex (ERC) and perirhinal cortex (PRC) as anterior parts, along with the posterior segment known as the parahippocampal cortex (PHC). However, recent research has detailed atlases based on cytoarchitectural characteristics and the hippocampus divided into four subregions: cornu ammonis (CA), dentate gyrus (DG), subiculum (SUB), and hippocampal–amygdaloid transition (HATA). This study aimed to explore the functional connectivity (FC) changes between these hippocampal subregions and the parahippocampal gyrus structures (ERC, PRC, and PHC) as well as between hippocampal subregions and various functional brain networks in schizophrenia. **Methods:** In total, 50 individuals with schizophrenia and 50 matched healthy subjects were examined using resting state functional magnetic resonance imaging (rs-fMRI). **Results:** The results showed alterations characterized by increases and decreases in the strength of the positive connectivity between the parahippocampal gyrus structures and the four hippocampal subregions when comparing patients with schizophrenia with healthy subjects. Alterations were observed among the hippocampal subregions and functional brain networks, as well as the formation of new connections and absence of connections. **Conclusions:** There is strong evidence that the different subregions of the hippocampus have unique functions and their connectivity with the parahippocampal cortices and brain networks are affected by schizophrenia.

## 1. Introduction

Schizophrenia is a chronic neurodegenerative psychiatric disorder with an early onset in young adulthood [1]. It presents with a wide range of symptoms, including hallucinations, delusions, and marked impairments in speech, behavior, and cognition. The heterogeneity of these symptoms, coupled with their early onset, makes schizophrenia a challenging disease, with difficulties in understanding its pathophysiological underpinnings, establishing its etiological factors, and defining clear diagnostic criteria. Further research is needed to better characterize the brain-based alterations associated with the disorder, which may support earlier diagnosis and more effective interventions [2].

Within the intricate landscape of the brain, the medial temporal lobe (MTL) occupies a pivotal role, encompassing components that are closely associated with cognitive and emotional functions [3]. Previous research indicates that people with schizophrenia exhibit impairments in their episodic memory function and structural abnormalities within the MTL. Additionally, prior studies have established correlations between a reduction in MTL volume and the observed deficits in episodic memory among individuals with schizophrenia [4,5]. Prominent regions within the MTL include the hippocampus together with its neighbor, the parahippocampal gyrus. The parahippocampal gyrus can be further divided into anterior and posterior segments, where the anterior section encompasses the entorhinal cortex (Brodmann area 28) and the perirhinal cortex (Brodmann area 35). The posterior part comprises the parahippocampal cortex (Brodmann area 36), and these regions play a crucial role in processes related to memory formation and spatial cognition [3].

Patients with schizophrenia often exhibit symptoms related to memory and cognitive function. The hippocampus and the PHC have been identified as pivotal components in the process of recollection in previous studies [5,6,7,8]. In contrast, the ERC and PRC have been associated with semantic [9,10] and item memory [5,8], all of which contribute to episodic memory. It is worth noting that individuals with schizophrenia exhibit deficiencies in both verbal and visual/spatial episodic memory [11,12,13].

However, it is important to note that the hippocampus is also a structurally and functionally complex region, further divisible into subregions based on the probabilistic cytoarchitectonic maps provided by the JuBrain anatomy toolbox. These subregions show differences in cell counts, neurons, and various chemical characteristics. They include the cornu ammonis (CA), the dentate gyrus (DG), the subiculum (SUB), and the hippocampal–amygdaloid transition area (HATA) [14,15,16]. Notably, the entorhinal cortex provides input to the DG, which then connects to the CA, with the SUB subregion being a primary target of the CA1 output before information is relayed back to the entorhinal cortex. Each of these subregions possesses distinct structural and functional characteristics, contributing differentially to memory and cognitive processes. A recent study indicates that they are affected differently in schizophrenia, with the exception of the HATA subregion, which was not included [17]. It is worth noting that HATA holds a unique position within the hippocampus–amygdala network, acting as a transitional area between these two critical structures. In studies exploring memory recall across the lifespan, HATA has been strongly associated with memory function, as well as its role in regulating fear, situational learning, and emotional memory [18]. Therefore, an exploration of the diverse hippocampal subregions may shed light on additional complexities of this complicated disorder. Information primarily moves from the perirhinal and parahippocampal cortices towards the entorhinal cortex and subsequently to the hippocampal formation. However, substantial information processing takes place within and between the cortices of the parahippocampal gyrus even before the hippocampal formation becomes engaged [3]

Rs-fMRI is a potent tool for investigating the intrinsic, low-frequency functional interactions between various brain regions during a state of rest, not only in schizophrenia but also in other disorders such as Alzheimer’s disease, major depressive disorder, and autism spectrum disorder, further demonstrating its broad utility [19]. This study focuses on the examination of brain connectivity in schizophrenia as observed through rs-fMRI. In recent research, the resting state FC of the hippocampus has been a subject of investigation. Notably, patients with schizophrenia have been observed to exhibit altered FC within this cortico-hippocampus network, specifically involving the PHC and PRC [20]. Furthermore, another study [21] investigated the connectivity of the hippocampus at a subregional level, focusing on anterior and posterior segments only. This study identified a complex pattern of connectivity alterations from the anterior and posterior hippocampal subregions to the default mode network (DMN), temporal, and occipital regions. The authors provided evidence and support for studying hippocampal subdivisions along the longitudinal axis in schizophrenia. Their results suggested that the abnormalities in the FC of the hippocampal subregions reflect deficits in episodic memory that may be implicated in the pathophysiology of schizophrenia [21]. It is worth noting that this study was task-based and limited to exploring the anterior and posterior subregions of the hippocampus. The underlying assumption in the present study is that the distinct anatomical characteristics of the four subregions of the hippocampus, each with its unique cytoarchitectonic features, are likely to show differences in FC.

Previous studies often treated the hippocampus as a single structure or divided it only into anterior and posterior segments [21]. This study hypothesizes that analyzing cytoarchitectonic subregions will uncover distinct patterns of altered functional connectivity (FC) in schizophrenia. Using resting state fMRI, we examined FC between four hippocampal subfields—cornu ammonis (CA), dentate gyrus (DG), subiculum (SUB), and the hippocampal–amygdaloid transition area (HATA)—and the parahippocampal cortices, as well as large-scale brain networks for patients with schizophrenia and healthy individuals. To our knowledge, this is the first study to include all four subregions, including HATA, in a comprehensive FC analysis. The overarching goal was to gain a clearer understanding of altered resting state FC patterns in schizophrenia and their potential relevance to the underlying neural mechanisms of the disorder.

## 2. Materials and Methods

### 2.1. Participants and Dataset

In total, 50 individuals diagnosed with schizophrenia and 50 matched healthy participants were selected for this research through community advertisements in the Los Angeles area. Subjects underwent comprehensive neurophysiological assessments and functional magnetic resonance imaging (fMRI) scans. The age range of participants was between 22 and 49, including 38 males and 12 females. Data were sourced from an openly accessible neuro organization, specifically the UCLA Consortium for Neuropsychiatric Phenomics LA5c Study [22,23], and obtained from the OpenfMRI database under accession number ds000030 (https://openneuro.org/datasets/ds000030/versions/1.0.0 (accessed on 22 July 2025)). This dataset includes age, gender, and subject-specific protocols while ensuring participant anonymity. Inclusion criteria required that participants have at least eight years of formal education and be native speakers of English or Spanish to ensure adequate cooperation in completing assessments. All participants tested negative for drugs of abuse, including cocaine, methamphetamine, morphine, THC, and benzodiazepines, through urinalysis. Exclusion criteria included contraindications for MRI scans (such as claustrophobia, metallic objects, inability to fit in the MR scanner, or pregnancy), medical illnesses, use of mood-altering medications on the day of scanning, insufficient vision for task stimuli, left-handedness, and a history of head injury with loss of consciousness. The approved procedures of the University of California Los Angeles Institutional Review Board granted all participants written informed consent. For more information, please visit the website at https://openneuro.org/datasets/ds000030/versions/1.0.0 (accessed on 22 July 2025). The present study was also approved by the Institutional Review Board of King Abdulaziz University (IRB File No. FAMS-EC2022-10 (21 April 2020)).

### 2.2. MRI Data Acquisition

Neuroimaging data were collected using a 3T Siemens Trio scanner (Siemens Medical Solutions, Erlangen, Germany) at the Ahmanson-Lovelace Brain Mapping Center, University of California, Los Angeles, CA, USA. The data acquisition included T1-weighted Anatomical MPRAGE images and rs-fMRI. Functional MR data was acquired using a T2*-weighted echo planar imaging (EPI) sequence with specific parameters: slice thickness = 4 mm, 34 slices, TR = 2 s, TE = 30 ms, flip angle = 90°, matrix = 64 64, and FOV. = 192 mm. High-resolution T1-weighted anatomical scans (MPRAGE) were obtained with the following settings: slice thickness = 1 mm, 176 slices, TR = 1.9 s, TE = 2.26 ms, matrix = 256 256, FOV = 250. During the resting state fMRI data collection, participants were instructed to remain relaxed and keep their eyes open for five minutes. No stimuli were presented, and participants were not required to respond to any tasks during the scan.

### 2.3. MRI Data Preprocessing

**Figure 1:**
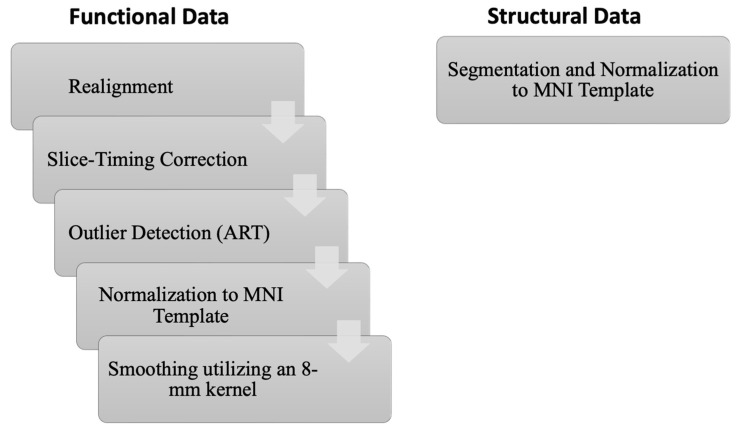
Schematic illustration of the preprocessing pipeline carried out by CONN and SPM12.

CONN toolbox (version 21.a) [24], in conjunction with Statistical Parametric Mapping software (SPM12, revision 7765, Wellcome Centre for Human Neuroimaging, London, UK) was employed to carry out preprocessing and statistical analysis. The standard preprocessing steps for rs-fMRI image sets encompassed adjustments for slice time correction, realignment of functional volumes, normalization to MNI templates using structural data, and identification of data outliers through implanted artifact detection tools (ART). Furthermore, a smoothing procedure was applied to the functional volumes, utilizing an 8 mm kernel (Figure 1). Additionally, temporal processing and removal of noise from the data were carried out to eliminate the influence of confounding factors and artifacts, including signals from white matter and cerebrospinal fluid (CSF), motion parameters, and scrubbing parameters from the original BOLD signal.

### 2.4. Selection of the Regions of Interest

The areas of interest chosen for this investigation consisted of the parahippocampal cortex (Brodmann area 36), perirhinal cortex (Brodmann area 35), and entorhinal cortex (ERC) as seeds. Additionally, four subregions of the hippocampus were considered targets, namely the cornu ammonis (CA), the dentate gyrus (DG), the subiculum (SUB) and the hippocampal–amygdaloid transition area (HATA). These specific hippocampal subregions and the ERC were identified through a cytoarchitectonic probabilistic anatomy map based on their cytoarchitectonic features [14,15,16] apart from the parahippocampal cortex and perirhinal cortex, which were located using the Brodmann Atlas (BA) [25,26,27,28] and defined based on Brodmann areas provided by CONN. The functional brain networks were also delineated in CONN. All the selected regions were normalized to the MNI space for all subjects. The selection of regions in this study was hypothesis driven. The parahippocampal cortices (ERC, PRC, PHC) were included due to their established roles in episodic memory and emotional regulation, which are frequently impaired in individuals with schizophrenia [5,7,8,9,10,11,12,13]. The four hippocampal subregions (CA, DG, SUB, HATA) were selected based on their distinct cytoarchitectonic profiles [14,15,16], which we hypothesized would reflect functional connectivity differences in schizophrenia.

### 2.5. Statistical Analysis

Statistical analysis comprised two levels. Initially, at the subject level, weighted general linear bivariate correlation models were computed, encompassing connectivity matrices between predefined regions of interest (ROIs) for each subject. These matrices represented Fisher-transformed correlation coefficients between pairs of ROI time series. Subsequently, at the second level, FC measures were calculated and contrasted using group-level statistical methods such as T-tests and, if appropriate, F-tests. This process aimed to identify and compare the rs-fMRI networks linked to each seed and each subregion of the hippocampus at the group level. Results were presented using a corrected false discovery rate (FDR) with a significance level of *p* < 0.05. This involved employing a multivariate statistics parametric (MVPA) omnibus test [24] based on cluster-level inferences. This approach considered groups or networks of interconnected ROIs, assessing both within- and between-network connectivity [29] This technique essentially employed a multivariate parametric analysis for all connections, yielding an F-statistical test map for each network pair [30]. The FDR cluster level referred to the anticipated proportion of false discoveries among all pairs of networks with comparable or greater effects across the entire set of functional connectivity network pairs. Opting for FDR rather than a family-wise error (FWE) was driven by FDR’s heightened sensitivity in controlling peaks while minimizing the risk of false positives [31]. These statistical methods were implemented using the CONN toolbox in MATLAB (version R2022a), which performs ROI-to-ROI and whole-brain voxel wise analyses using SPM-based general linear modeling. The MVPA test enhances sensitivity by identifying multivariate patterns of group-level differences across ROIs [24]. For more comprehensive information, please refer to the above papers or to the CONN website (https://web.conn-toolbox.org (accessed on 22 July 2025)).

## 3. Results

**Figure 2:**
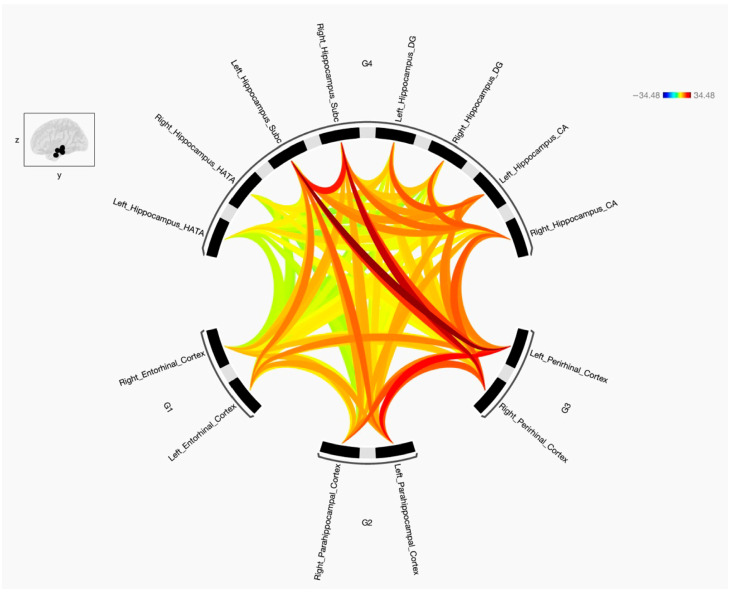
The FC among the entorhinal cortex (ERC), perirhinal cortex (PRC), and parahippocampal cortex (PHC), as well as four sub-regions of the hippocampus, was examined in healthy subjects (*n* = 50). The parahippocampal cortex includes regions in both the right and left hemispheres (6 regions), along with four hippocampal sub-regions (8 sub-regions). The red lines indicate positive connectivity, while blue indicates negative connectivity—although no significant negative connections were observed in this figure. The color intensity is proportional to statistical strength. The T-bar shown in the top right-hand corner represents the T-statistic values. All connections shown are significant at FDR-corrected *p* < 0.05.

**Figure 3:**
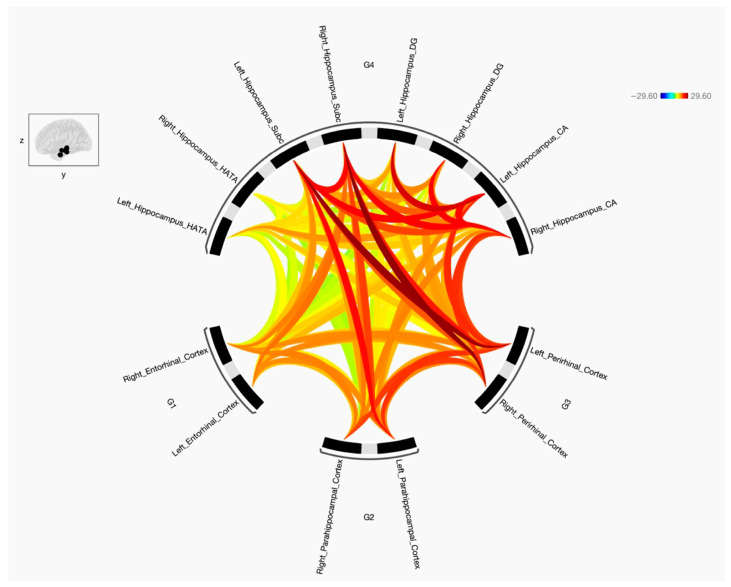
The FC among the entorhinal cortex (ERC), perirhinal cortex (PRC), and parahippocampal cortex (PHC), as well as four sub-regions of the hippocampus, was examined in subjects with schizophrenia. The parahippocampal comprises regions in both the right and left hemispheres (6 regions), along with four hippocampal sub-regions (8 sub-regions). The red lines indicate positive connectivity, while blue indicates negative connectivity—although no significant negative connections were observed in this figure. The color intensity is proportional to statistical strength. The T-bar shown in the top right-hand corner represents the T-statistic values. All connections shown are significant at FDR-corrected *p* < 0.05.

This study was undertaken to explore the FC and the strength of the connections between the parahippocampal gyrus, encompassing its anterior subdivisions ERC and PRC and its posterior segment PHC, and four specific targeted subregions of the hippocampus (CA, DG, SUB, and HATA). Additionally, it aimed to determine disparities in FC patterns between the preidentified seeds and targets in the left and right cerebral hemispheres between two distinct groups, namely individuals diagnosed with schizophrenia and those characterized as healthy subjects. The results indicate that the three parahippocampal cortex regions (ERC, PRC, and PHC) exhibit connections with the four hippocampal subregions including CA, DG, SUB, and HATA, with either positive or negative connections based on statistical variations for both groups. Figure 2 illustrates the FC between the seeds of the parahippocampal cortex and the specified hippocampal subregions for healthy subjects, while Figure 3 displays the same for individuals with schizophrenia. It is worth noting that the FC between the predetermined parahippocampal gyrus seeds (ERC, PRC, and PHC) increased in the group with the disorder. Moreover, disruptions were observed in the connectivity between ERC, PRC, and PHC and the hippocampal subregions (CA, DG, SUB, and HATA) in individuals with schizophrenia, resulting in either increased or decreased FC.

### 3.1. Changes in Functional Connectivity in the Parahippocampal Gyrus Cortices

Overall, our study revealed only an increase in FC within the seed regions when comparing individuals with schizophrenia to healthy subjects. When looking at the diseased group, the same cortices in both hemispheres exhibited increased FC, specifically the right and left entorhinal cortices (ERCs) and the right and left parahippocampal cortices (PHCs), except for the perirhinal cortices. Additionally, the results showed that the right PRC displayed a stronger connection with the right PHC and bilateral ERCs, while the left PRC showed a stronger connection solely with the right ERC. Furthermore, the right and left PHCs demonstrated increased FC with bilateral ERCs. Lastly, the right ERC exhibited a stronger connection with the left PHC.

### 3.2. Resting State Functional Connectivity Between ERC, PRC, PHC and Hippocampal Subregions

When comparing individuals with schizophrenia and healthy subjects and analyzing the data from Figure 1 and Figure 2, the diseased group displayed alterations in resting state FC between the parahippocampal cortex, perirhinal cortex, entorhinal cortex, and various subregions of the hippocampus. Specifically, the right parahippocampal cortex exhibited decreased FC with the left hemisphere hippocampal subregion, including left SUB, left CA, left DG, and left HATA. In contrast, the left parahippocampal displayed increased FC with the right SUB subregion of the hippocampus but decreased FC with the bilateral CA, bilateral DG, and bilateral HATA subregions. When looking at the other subregions, the right perirhinal cortex showed increased FC with the right DG and right CA subregions in individuals with schizophrenia, along with decreased FC to the left SUB, left CA, and right HATA subregions. Conversely, the left perirhinal cortex demonstrated increased FC with the bilateral CA, bilateral DG, and bilateral HATA subregions in the diseased group when compared to the control group. Moreover, the right entorhinal cortex displayed decreased FC with several subregions, including the left SUB, left CA, left DG, and right HATA subregions. In contrast, the left entorhinal cortex exhibited increased FC with the right SUB, right CA, and bilateral HATA subregions but decreased FC with the left SUB, left CA, and bilateral DG subregions of the hippocampus.

### 3.3. Functional Connectivity with the Default Mode Network (DMN)

**Figure 4:**
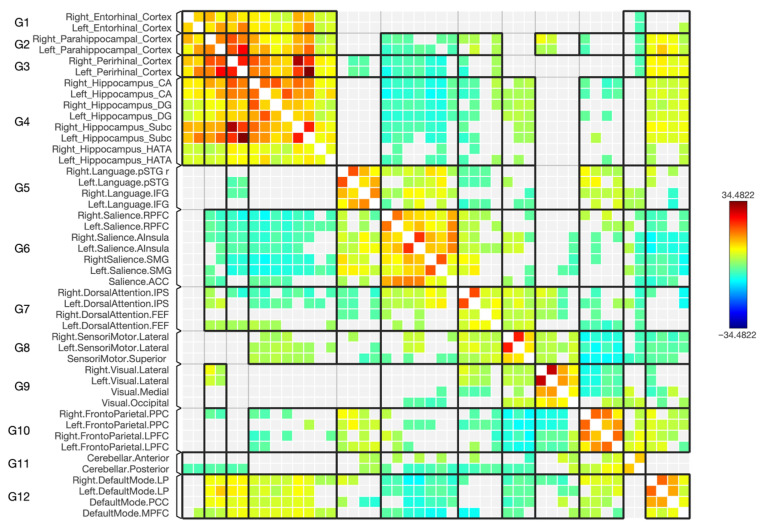
The connectivity matrix of the hippocampus subregions and parahippocampus cortices, along with the selected functional brain networks for the control subjects (*n* = 50). The color bar on the right reflects T-statistic values: red indicates stronger positive FC, and blue indicates stronger negative FC. The results have been thresholded at FDR-corrected *p* < 0.05.

**Figure 5:**
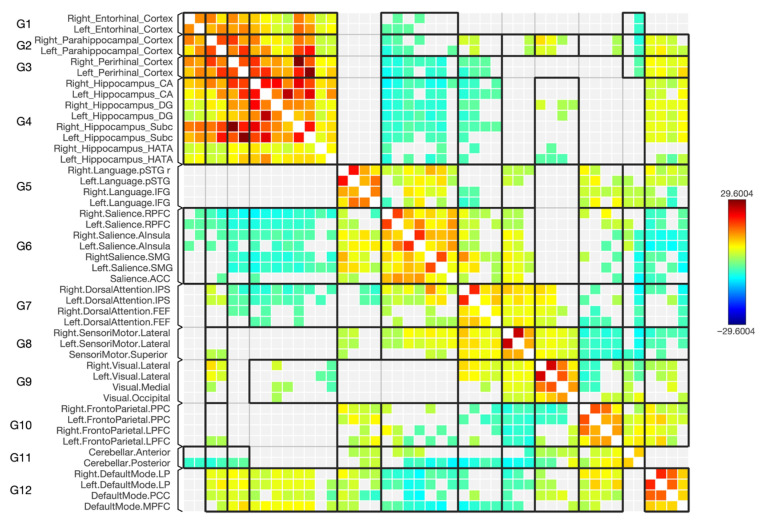
The connectivity matrix of the hippocampus subregions and parahippocampus cortices, along with the selected functional brain networks for schizophrenia (*n* = 50). The color bar on the right reflects T-statistic values: red indicates stronger positive FC, and blue indicates stronger negative FC. The results have been thresholded at FDR-corrected *p* < 0.05.

In general, as depicted in the matrices in Figure 4 and Figure 5, the majority of the hippocampal subregions exhibited changes in the negative connectivity with the regions defining the DMN and an absence as well as new connections when comparing patients with schizophrenia with healthy subjects. However, in individuals with schizophrenia, the left CA, right CA, left DG, right DG, and left SUB subregions displayed a stronger negative connection with the medial prefrontal cortex (MPFC) area within the DMN. The right HATA subregion showed no connection with MPFC, whereas this was connected in healthy subjects. An absence of connection with the posterior cingulate cortex (PCC) area was displayed by the left CA subregion for the diseased group, together with a decrease in the connection to PCC by the right CA, left DG, right SUB, and left SUB. Additionally, a new connection from the left HATA subregion to the PCC area was shown, which was absent in the controls. For the bilateral lateral parietal (LP) area, a stronger connection was noticed with the right DG subregion and an absence of connection along the right LP in patients with schizophrenia with the right and left HATA subregions, whereas the left HATA subregion exhibited simultaneously a new connection with the left LP area within the DMN, which was absent in the controls.

### 3.4. Functional Connectivity with the Frontoparietal Network

As shown in the matrices in Figure 4 and Figure 5, many of the hippocampal subregions showed an absence of connections within the regions defining the frontoparietal network when comparing patients with schizophrenia with healthy subjects. Specifically, for the diseased group, the lateral prefrontal cortex (LPFC) in both hemispheres had a missing connection with the right and left CA subregions. While the left DG and left HATA had a missing connection with only the right LPFC, in both situations it displayed a negative connection in the control group. Moreover, in schizophrenia, the right posterior parietal cortex (PPC) also had no connections with multiple subregions: left CA, right CA, and left DG. Nonetheless, the left SUB subregion had a missing connection as well, but with the left PPC.

### 3.5. Functional Connectivity with the Sensorimotor Network

As shown in the matrices in Figure 4 and Figure 5, most of the hippocampal subregions displayed an absence of connections with the regions defining the sensorimotor network for the diseased patients, while there was a negative connection for the control group. However, the bilateral CA, bilateral DG, and bilateral SUB subregions showed a missing connection with the superior cortex sub-area within the sensorimotor network. Additionally, the left lateral cortex showed a missing connection with bilateral CA, bilateral DG, bilateral SUB, and left HATA subregions, whereas the right lateral cortex exhibited the same result, but with left CA and bilateral DG subregions only for the diseased group.

### 3.6. Functional Connectivity with the Dorsal Attention Network

In general, as depicted in the matrices in Figure 4 and Figure 5, most of the hippocampal subregions exhibited changes in the negative connectivity with the regions defining the dorsal attention network and an absence as well as new connections when comparing patients with schizophrenia with healthy subjects. More precisely, when looking at patients with schizophrenia, a lower strength of negative connectivity was observed along the left frontal eye field (FEF) and bilateral CA subregions, together with an absence of a connection with the right DG and left HATA sub-fields and a new connection with the right SUB. Regarding the right FEF, patients with schizophrenia displayed new connections with the left CA and the right SUB hippocampal subregions. Additionally, in schizophrenia, the right intraparietal sulcus (IPS) had a new connection with the right DG, while the left IPS showed a new connection with the right SUB and an absence of one with the left SUB.

### 3.7. Functional Connectivity with the Salience Network

As shown in the matrices in Figure 4 and Figure 5, some of the hippocampal subregions displayed either an absence of connection or a new connection with salience network sub-fields when comparing patients with schizophrenia with healthy subjects. However, within the diseased group, the only decrease in connectivity was noticed between the left anterior insular cortex (AInsula) and the bilateral rostro-lateral prefrontal cortex (RPFC) with the right DG hippocampal subregion. In contrast, there was a missing connection in the diseased group with the anterior cingulate cortex (ACC) from the left CA and bilateral DG. Additionally, there was a missing connection between the left AInsula and the left CA, left RPFC, and bilateral HATA subregions. On the other hand, new connections in the diseased group were observed between the right AInsula and the left HATA, right RPFC and right HATA.

### 3.8. Functional Connectivity with the Visual Network

As shown in the matrices in Figure 4 and Figure 5, some of the hippocampal subregions exhibited new connections with the sub-fields of the visual network in the diseased group, which were absent in the control group. Although the bilateral DG hippocampal subregions shared new connections with the medial visual area within the network, the right DG had additional new connections with the occipital and right lateral cortices. In contrast, the left HATA showed new connections with the medial, right lateral, and left lateral areas within the visual network, but for the right HATA subregion, a new connection with the left lateral sub-field was observed.

## 4. Discussion

### 4.1. Changes in the Connectivity Between Parahippocampal Cortices and Hippocampal Subregions

The current study investigated differences in intrinsic FC between patients with schizophrenia and the control group. Our research focused on specific brain regions, specifically the structures within the parahippocampal gyrus, including the ERC and PRC anteriorly and the PHC posteriorly [3]. These structures served as the seeds of our study. Additionally, we examined four specific cytoarchitectonically defined subregions within the hippocampus, namely CA, DG, SUB, and HATA [14,15,16].

Our primary assumption was that due to the distinct cytoarchitectural characteristics of these subregions, their functional connections with parahippocampal cortices and various brain networks might exhibit variations. In summary, we found differences in connectivity among these hippocampal subregions in patients with schizophrenia when compared with controls. These differences were characterized by either increased or decreased FC, and the extent of these alterations was correlated with statistical strength.

The hippocampus, a key player in emotional regulation, stress responses, and declarative memory, has long been recognized as a central region in the study of schizophrenia [32,33,34]. Extensive research has firmly established findings such as reduced hippocampal volume [35] decreased episodic memory performance [36] and physiological irregularities [37] in patients with schizophrenia compared with control groups. However, most previous brain-imaging studies related to schizophrenia have mainly focused on the entire hippocampus or its anterior and posterior segments [38]. The presumption has been that the anterior part is linked to anxiety-related functions, while the posterior segment is associated with spatial navigation and memory-related behaviors [39]. Nonetheless, the hippocampus is a structurally complex region comprising four distinct subfields [14,15,16] each contributing differently to memory and cognitive processes [40]. Recent research has shown that schizophrenia, bipolar disorder, and major depressive disorder, which share etiological and pathophysiological characteristics, have varying impacts on these hippocampal subregions, including CA, DG, and SUB. These findings may serve as potential biomarkers for each disease, reflecting intrinsic characteristics relevant to these psychiatric disorders. In particular, a decreased FC between the CA subregion and the caudate nucleus is a unique feature that distinguishes schizophrenia from bipolar disorder and major depressive disorder [17]. These findings align with the main objective of our study, emphasizing the importance of studying the hippocampus at the subregional level.

Among all hippocampal subregions, the most robust and consistent alteration observed in our study was the decreased FC involving the left CA subregion. This was evident across nearly all seed regions except the left PRC, reinforcing the importance of the left CA as a key hub of dysfunction in schizophrenia. This finding complements recent research identifying CA dysconnectivity as a feature distinguishing schizophrenia from other psychiatric disorders [17] and adds to a growing body of literature implicating the CA1/CA subregion in the pathophysiology of psychosis, memory dysfunction, and structural vulnerability [38,41,42,43]. Several studies have consistently demonstrated that the CA1 subregion undergoes specific volume reduction in the early stages of schizophrenia [41] which has been associated with worsening symptom severity over time [42] and with higher antipsychotic medication doses [43]. A comprehensive review further highlights a consistent pattern of reduced CA1 volume alongside increased CA1 activity in schizophrenia, suggesting its potential as a predictive biomarker for psychosis risk. Moreover, studies have linked hippocampal subregion volumes to both declarative memory and symptom severity in schizophrenia [38]. The increased CA1 activity may help explain additional findings from our study, in which the left PRC exhibited increased FC with bilateral CA regions, the right PRC showed increased FC with the right CA, and the left ERC exhibited increased FC with right CA. Furthermore, increased FC within bilateral PRC, L PHC, and L ERC and some hippocampal subregions can be explained by the compensatory hypothesis, which was supported by the findings of increased FC in some brain areas among genetically predisposed young individuals who are cognitively healthy but at risk of developing neuro-degenerative conditions [44]. This compensatory mechanism has been proposed as a way to account for the decrease in interhemispheric inhibition in another study [45] and may also explain the increased FC found in schizophrenia in the current study. An alternative interpretation posits that this increased FC represents a pathological state that could potentially lead to further brain damage, as indicated in some studies [46,47]. However, this hypothesis needs to be further investigated in schizophrenia.

It has been hypothesized that HATA is highly associated with memory function. In addition, it has a role in fear regulation, situational learning, and emotional memory, and atrophy of this subregion may be related to a decline in adaptability to new environments in older people with cognitive frailty, but further research is required to validate this hypothesis [18]. Accordingly, this study uncovers information about this rarely investigated subregion and its association with the disease. Decreased FC in this region is seen in the right PRC, bilateral PHC cortices, and left ERC. Almost all the seeds showed decreased FC, and one study conducted in 2020 showed a reduction in the volume of the CA1, HATA, fimbria, and DG subregions in patients with schizophrenia with a history of violence, which indicated that the atrophy of these volumes could be associated with the risk of violence in individuals with schizophrenia. Moreover, these hippocampal subregions are important for regulating emotions, having control over instrumental behaviors, and regulating fear [48]. Since the HATA subregion has been hypothesized to be involved in fear regulation [18], it suggested from our results that decreased FC with this subregion might be linked to such an issue with patients with schizophrenia.

Previous research has established that CA and DG subregions play similar roles in encoding new associations with novel information [49] and are implicated in the neuroimaging mechanisms related to psychiatric disorders [17]. Our study’s findings align with this hypothesis. Specifically, they demonstrate increased FC between the left PRC and bilateral CA, DG, and HATA subregions. Conversely, the left PHC displayed decreased FC with bilateral CA, DG, and HATA sub-areas. These findings support the notion that CA and DG share roles in associative encoding and their relevance to psychiatric disorders. While the majority of our results align with the roles of CA and DG in psychiatric disorders, there was an exception observed with the ERC. Specifically, the left ERC exhibited increased FC with R CA but decreased FC with L CA and bilateral DG subregions. Notably, a systemic review article has suggested that impairments in the DG-CA3-CA1-SUBC circuit may play a role in the development of psychosis, with the extent of changes and their impact varying depending on the stage of the illness [38]. Our study further contributes to this understanding by providing insights into how this impairment may manifest in individuals with the disease. These disrupted connectivity patterns, particularly involving CA, DG, and HATA, might help explain the memory and emotional symptoms frequently observed in schizophrenia [18,38,48] although further research is needed to confirm these functional relationships.

The SUB receives direct signals from the hippocampal CA1 region and transmits information to multiple areas in the cortex and subcortex. It plays a vital role in structuring the information flow from the hippocampus and possesses a distinct function in information processing [50]. Recent research has further delineated the specific functions of the SUB subregion, highlighting its role in memory retrieval, while the CA subregion is involved in both memory encoding and retrieval based on task-based fMRI experiments [51]. Our study contributes to this understanding by uncovering changes in the FC associated with the SUB in patients with schizophrenia. Specifically, we observed a decreased FC with the SUB in the right PRC, right PHC, and bilateral ERCs. Conversely, we noted an increased FC from two seed regions—the left PHC and left ERC. These findings shed light on the alterations that may underlie the impairment in memory retrieval that is observed in individuals with schizophrenia.

Importantly, the ERC is connected directly to the hippocampal subregions, whereas the PRC and PHC are positively connected to the hippocampal subregions in disease and control directly or indirectly via the ERC [52,53]. Cortical information primarily flows from the parahippocampal and perirhinal cortices towards the entorhinal cortex, eventually engaging the hippocampal formation. Nevertheless, significant information processing occurs within the cortices of the parahippocampal gyrus even before the hippocampal formation becomes active [3]. One study evaluated the hippocampus and adjacent parahippocampal cortices volumes in schizophrenia and concluded that the discovery of reduced volumes in the medial temporal lobe, without specific localization, holds significant implications for investigating how the hippocampus and adjacent cortical areas function in schizophrenia [54]. In our study, we found that, in general, all parahippocampal cortices exhibited functional connections to the hippocampus at the subregional level in schizophrenia, displaying a diverse pattern of connectivity, with FC alterations including both increased and decreased FC. This can aid in our comprehension of the disease’s neural mechanisms for these particular regions. Another study further emphasized the significance of investigating the parahippocampal cortices in schizophrenia [55]. The authors concluded that the parahippocampal cortices are involved in the episodic memory deficits observed in schizophrenia, since they observed reduced connectivity within these cortices. In contrast, our results revealed increased FC within ERC, PRC, and PHC. However, FC decreased in nearly all hippocampal subregions. Furthermore, the primary cortical input to the hippocampus comes from both the medial entorhinal cortex (MEC) and the lateral entorhinal cortex (LEC). It is widely accepted that the MEC conveys spatial information to the hippocampus, while the LEC conveys non-spatial information [56]. According to the findings of a recent study, the entorhinal cortex can be subdivided into two distinct regions. The “medial entorhinal cortex” primarily forms connections with the parahippocampal cortex, which plays a role in processing visual scenes. On the other hand, the “lateral entorhinal cortex” exhibits particularly strong connections with the perirhinal cortex, which is involved in object memory. Additionally, these two subregions of the entorhinal cortex are linked to different segments of the hippocampus [57]. There is a suggestion that the heightened FC between the ERC and both the PRC and PHC could be associated with visual processing impairment observed in individuals with schizophrenia. However, our study has limitations since it focuses on the overall findings of the ERC and does not delve into the subregional level. This could be explored in future research. Based on the background of the entorhinal cortex, our study revealed that both sides of the entorhinal cortex exhibited reduced FC with specific subregions, including the left SUB and left CA subregions. Additionally, the left entorhinal cortex displayed reduced FC with both sides of the DG subregions, while the right entorhinal cortex showed reduced FC with the left DG and right HATA subregions.

A structural MRI study found that individuals with schizophrenia had a smaller left ERC compared to healthy subjects. Notably, among patients with schizophrenia, those without delusions exhibited smaller ERC sizes than those with delusional psychotic disorders. This suggests a significant association between the ERC pathology and positive symptoms, in particular delusions. The decline in ERC functions, including recognizing novelty, forming associations, and managing various forms of memory, may underlie its connection to psychotic conditions and delusions [58]. A literature review further supports the link between structural and functional changes in MTL structures and various neuropsychiatric manifestations of schizophrenia [59]. Additionally, many structural neuroimaging studies have revealed a significant correlation between reductions in hippocampal volume and impaired episodic memory performance in individuals with schizophrenia [60,61].

Finally, the findings of this study regarding changes in FC between the parahippocampal cortices and hippocampal subregions in schizophrenia are of considerable importance. They provide clear evidence that, in addition to the cytoarchitectural distinctions among these four hippocampal subregions, there are also FC differences that deserve careful consideration. When examining alterations in FC within these hippocampal subregions in conjunction with the adjacent parahippocampal cortices, it becomes evident that employing more detailed anatomical atlases for precise investigations is crucial. Such an approach can contribute significantly to our understanding of the neural mechanisms involved in the development of neuro-degenerative diseases.

### 4.2. Changes in the Hippocampal Subregion’s Connectivity with Functional Brain Networks

Additionally, for a better understanding of the hippocampal subdivisions’ FC with multiple brain networks, the rs-FC was assessed. There were changes in the connectivity with the various brain networks, missing connections, and forming of new functional negative connections. The majority of the networks analyzed in this study exhibited weak negative connections with hippocampal subregions, as indicated by low statistical significance. However, it is important to note that weak negative connections were also present in the control group, with some changes seen in the diseased group. However, the detection of negative FC or the presence of anticorrelated signals in rs-fMRI is a subject that continues to be a matter of debate and active research within the field of functional brain imaging. Anticorrelations indicate negative associations between the time series activity in two brain regions or networks, suggesting that when one region’s activity increases, the other tends to decrease [62,63]. Although the interpretation of these anti-correlated signals poses challenges, researchers have several possible explanations, encompassing neural origins, artifacts, and combinations of neural and non-neural factors [62,64]. These findings warrant more extensive analysis, but our study’s primary focus did not include investigating them in detail. This is primarily because of the intricate nature of characterizing and comprehending anticorrelation signals in rs-fMRI. First, the DMN comprises brain areas such as the posterior cingulate cortex (PCC), medial prefrontal cortex (MPFC), inferior parietal region, and parahippocampal gyrus [65]. This network has been recognized for its significant involvement in self-referential and reflective processes [66,67], as well as its role in focusing on both internal and external stimuli [65,67,68]. Irregular activity and FC in the DMN have been documented in schizophrenia, but the findings are inconsistent: some studies report an increase in FC [69,70,71] some report a decrease [72,73,74], and others find both increases and decreases [75,76]. The inconsistent results could be due to the varied neuroimaging methods and diverse methods of categorizing hippocampal regions employed in these studies [77]. Our study utilized the ROI-to-ROI analysis method to explore the differences in FC in various brain networks with the disease. For the DMN, there was an increase in the FC from bilateral CA, bilateral DG, and left SUB subregions to the MPFC area within the DMN. A task-based and resting state study reported that patients with schizophrenia and their relatives had hyperactivation and hyperconnectivity in MPFC that eventually could play a role in thought disturbances in schizophrenia and a risk factor for developing the condition [78]. Additionally, alterations in resting state connectivity were detected in the connections between the hippocampus and areas within the DMN, such as the medial prefrontal cortex, PCC, and precuneus [79,80,81] which confirms the results of this study at the subregional level. Here, a decreased FC was detected from right CA, left DG, and bilateral SUB to PCC. An absence of connection with the disease was also detected from left CA to PCC. This study explained at the subregional level the alteration that occurred in the DMN. Moreover, most of the hippocampal subregions had missing connections to sensorimotor, frontoparietal, and salience networks that were present in the control group. Deficit in FC has been observed between the posterior hippocampus and the ACC area within the salience network and reported in many studies [79,82,83]. Our study confirms that the existence of missing connections is specifically observed in the left CA and bilateral DG subregions to the ACC region within the salience network. Notably, these connections were present in the control group. A malfunction in the salience network can trigger a series of occurrences that lead to the manifestation of psychotic clinical symptoms [84]. This study shed light on the dysfunction seen in this network with hippocampal subregions. However, it may be noted that, in the control group, there was no connection with the areas defining the visual network, while some subregions showed new connections with this network, including bilateral DG and bilateral HATA subregions. In a study that focused on capturing symptoms, one individual with schizophrenia experiencing visual hallucinations displayed brain activity in higher visual regions that aligned with the content of their hallucinations, including faces, bodies, and scenes. Additionally, there was activity in the hippocampus, possibly linked to the retrieval of visual images from memory [85]. This could be the potential explanation for these connections seen in hippocampal subregions.

Investigating neural networks and their interactions, rather than focusing solely on individual brain regions, is important for understanding the complexity of schizophrenia and related disorders. It emphasizes the importance of this approach in gaining insights into the neural mechanisms underlying the condition and improving our understanding of brain connectivity in neurodevelopmental disorders.

## 5. Conclusions

Our identification of the increased and decreased FC in patients with schizophrenia between the prominently affected brain regions, namely the parahippocampal gyrus structures and the hippocampal subregions, holds the potential to shed light on the neural mechanisms underpinning this disorder. Additionally, it enriches our overall understanding of resting state FC in the brain and its significance within neurodevelopmental disorders.

While rs-fMRI studies offer valuable insights into the development of schizophrenia, they cannot directly establish causal relationships between the various factors influencing its functioning. Furthermore, the findings from these studies may exhibit variability due to differences in sample characteristics, imaging techniques, and data analysis methods. Further investigations are warranted to delineate the role of distinct hippocampal subregions and their interconnections with parahippocampal cortices in this condition.

It is important to recognize that the hippocampus is a complex brain structure with multiple sub-regions, and that its functions extend beyond those mentioned earlier. Therefore, additional research is necessary to elucidate comprehensively the specific contributions of the preidentified seeds and targets to the intricate neurobiology of schizophrenia. The integration of findings from various imaging modalities, encompassing functional, structural, and diffusion tensor imaging (DTI) scans, will continue to expand our knowledge of the role of this region in schizophrenia. Future studies would benefit from incorporating longitudinal data, examining sex-based differences, and integrating multimodal approaches to further clarify the clinical implications of these connectivity alterations and their evolution across different stages of the disorder.

## 6. Limitations

One limitation of this study is our use of an open-access dataset, which imposes constraints on the available information for our statistical analysis. Additional data, particularly concerning potential confounding variables, such as psychiatric medications that may impact brain function, would enhance the depth of our investigations. For instance, further research involving drug-naive patients is needed to clarify the mechanisms by which different hippocampal subregions are involved. Additionally, our sample encompasses a wide age range (22 to 49 years old), and age itself can affect brain function. To mitigate the influence of age on our results, age-matched healthy controls were employed.

Additionally, the lack of specific analysis regarding gender differences in hippocampal subregions. Recent research suggests that males and females may exhibit different connectivity patterns [77], which could influence the functional alterations observed in schizophrenia. Future studies should consider these gender-specific variations to provide a more comprehensive understanding of the disease.

In conclusion, our research is based on a cross-sectional study. A longitudinal study is now required to gain a deeper understanding of the pathophysiological mechanisms that apply across different disorders.

## Data Availability

All data used in the study are available on the website OpenNEURO https://openneuro.org/datasets/ds000030/versions/1.0.0 (accessed on 22 July 2025).

## Abbreviations

ERC: Entorhinal cortex. PHC: Parahippocampal cortex. PRC: Perirhinal cortex. CA: Cornu ammonis. DG: Dentate gyrus. SUB: Subiculum. HATA: hippocampal–amygdaloid transition area. FC: functional connectivity. fMRI: Functional magnetic resonance imaging. rs-fMRI: resting state functional magnetic resonance imaging. MTL: medial temporal lobe. AInsula: anterior insular cortex.
